# Integrated methylome and transcriptome analysis unravel the cold tolerance mechanism in winter rapeseed(*Brassica napus L.*)

**DOI:** 10.1186/s12870-022-03797-1

**Published:** 2022-08-26

**Authors:** Guoqiang Zheng, Xiaoyun Dong, Jiaping Wei, Zigang Liu, Ali Aslam, JunMei Cui, Hui Li, Ying Wang, Haiyan Tian, Xiaodong Cao

**Affiliations:** 1 State Key Laboratory of Aridland Crop Sciences, Lanzhou, China; 2 College of Agronomy, Gansu Agricultural University, Lanzhou, China; 3 Affiliation Faculty of Agriculture and Veterinary Sciences, Superior University, Lahore, Pakistan

**Keywords:** Winter rapeseed, Freezing stress, DNA methylation, Freezing tolerance, Transcriptome

## Abstract

**Background:**

Cytosine methylation, the main type of DNA methylation, regulates gene expression in plant response to environmental stress. The winter rapeseed has high economic and ecological value in China's Northwest, but the DNA methylation pattern of winter rapeseed during freezing stress remains unclear.

**Result:**

This study integrated the methylome and transcriptome to explore the genome-scale DNA methylation pattern and its regulated pathway of winter rapeseed, using freezing-sensitive (NF) and freezing-resistant (NS) cultivars.The average methylation level decreased under freezing stress, and the decline in NF was stronger than NS after freezing stress. The CG methylation level was the highest among the three contexts of CG, CHG, and CHH. At the same time, the CHH proportion was high, and the methylation levels were highest 2 kb up/downstream, followed by the intron region. The C sub-genomes methylation level was higher than the A sub-genomes. The methylation levels of chloroplast and mitochondrial DNA were much lower than the *B. napus* nuclear DNA, the SINE methylation level was highest among four types of transposable elements (TEs), and the preferred sequence of DNA methylation did not change after freezing stress. A total of 1732 differentially expressed genes associated with differentially methylated genes (DMEGs) were identified in two cultivars under 12 h and 24 h in three contexts by combining whole-genome bisulfite sequencing( and RNA-Seq data. Function enrichment analysis showed that most DMEGs participated in linoleic acid metabolism, alpha-linolenic acid metabolism, carbon fixation in photosynthetic organisms, flavonoid biosynthesis, and plant hormone signal transduction pathways. Meanwhile, some DMEGs encode core transcription factors in plant response to stress.

**Conclusion:**

Based on the findings of DNA methylation, the freezing tolerance of winter rapeseed is achieved by enhanced signal transduction, lower lipid peroxidation, stronger cell stability, increased osmolytes, and greater reactive oxygen species (ROS) scavenging. These results provide novel insights into better knowledge of the methylation regulation of tolerance mechanism in winter rapeseed under freezing stress.

**Supplementary Information:**

The online version contains supplementary material available at 10.1186/s12870-022-03797-1.

## Introduction

Cold stress mainly includes chilling stress (above 0 °C) and freezing stress (below 0 °C). Freezing stress is one of the most unfavorable abiotic stresses in Northwestern China from late autumn to early spring, seriously affecting the growth, development, quality, and productivity of crops, and even restricting the geographical distribution of plant species, making this region relatively single species. Freezing stress damage includes multiple aspects, for example, cold, dehydration, osmotic, and mechanical stresses, which are more severe than chilling stress damage [1, 2].To cope with freezing injury, plants have evolved various adaptive mechanisms to respond and survive to low freezing conditions, including changes in physiological, biochemical, cellular, and molecular changes [3, 4]. Freezing tolerance is a necessary trait for crops to withstand low temperatures, especially in overwintering crops [5]. Some research showed that the freezing tolerance of plants is associated with cold acclimation, while DNA methylation plays a vital role [6]. At the same time, these studies showed that epigenetic mechanisms form stress memory, which offspring can inherit [7].

DNA methylation, one of the hallmarks of epigenetic mechanisms, plays an important role in plant adaptation to abiotic stresses by regulating transcriptional activities of the stress-response genes [8, 9]. A previous study showed that cold- induced demethylation in maize is involved in many processes, such as hormone regulation, cold response, photosynthesis, and transposon activation [10]. In rice, DNA methylation level was down-regulated in response to chilling stress, and differentially methylated genes were involved in regulating transcription factors families' [11]. In addition, the DNA methylation patterns changed in *Celtis bungeana* after chilling and freezing stresses [5]. In *Brassica rapa*, cold acclimation altered DNA methylation patterns and confers tolerance to heat and increases growth rate [12]. In tea plants significant loss of DNA methylation is found at their promoter and gene body regions after chilling stress; DNA methylation in the promoter positively regulates the gene expression, and negatively regulates the gene expression of gene body regions [13].

The rapeseed (*Brassica napus* L.) is an important oil-yielding seed crops all over the worldwide, with the largest planting area and relatively high yield and oil content. The major area of winter *B.napus* is the Yangtze River basin, which belongs to semi-winter rapeseed and more than 80% of china’s total area of rapeseed [14]. In a decade year, we have successfully cultivated the freezing-resistant cultivar NS, which can survive normally in winter at the -26℃ in most northwestern areas and contain abundant freezing-resistant genes, originating from the distant hybridization and cold acclimation between *B.napus* and *B. Rapa* [15, 16]. It is of great significance to continuously increase the planting areas of winter rapeseed in northern China for exploiting its economic value and ecological value, whereas, to expand the planting areas of winter rapeseed, we must understand molecular genetic mechanism of cold resistance and breed more winter rapeseed cultivars with strong cold tolerance. In recent years, the DNA methylation was the research hotspot on plant response to abiotic stresses, especially in cold stress. However, the epigenetic mechanism of how DNA methylation regulates genes to elevate cold tolerance of winter rapeseed are still little known.

The study first investigated the changes in methylation patterns of a pair of resistant and susceptible winter rapeseed cultivars after freezing stress. Meanwhile, the methylome and transcriptome were used to identify differentially methylated regions (DMRs), differentially methylated genes (DMGs), and differentially expressed genes associated with differentially methylated genes (DMEGs) between the two cultivars. Subsequently, the main metabolic pathways enriched in DMEGs involved in freezing stress were explored. These results will enable us to understand better the methylation-participated molecular regulatory network in winter rapeseed under freezing stress.

## Results

### Genome-wide DNA methylation profiles of NS and NF

Six genomic DNA libraries were constructed with the leaves of cultivars NS and NF to study the genome-wide DNA methylation pattern of winter rapeseed under freezing treatment.More than 192 million raw reads per DNA library samples were generated by whole-genome bisulfite sequencing (WGBS). After removal of related adapters, low-quality reads, poly-A and Ns-containing reads, 219 million, 190 million, 199 million, 221 million, 243 million, and 288 million were collected in six libraries (Table 1), of which 85.82% (NSt0), 86.38% (NSt1), 85.46% (NSt2), 86.96% (NFt0), 88.92% (NFt1), 86.87% (NFt2) were uniquely mapped to the *Brassica napus* L reference genome (GCA_000686985.2), respectively. The average sequence depths of all libraries were more than 25.35 fold, and the effective coverage rates of all chromosomes were between 50–60% (Supplementary Table S1). However, practically cytosines conversion rate for all libraries were close to 99%, indicating a high bisulfite sequencing conversion rate in this study.

**Table 1:** Whole genome DNA bisulfite sequencing data in two rapeseed cultivars

Samples	raw reads	clean Reads	Mapped Reads	Mapped Ratio(%)	Sequence Depth	ratio(%)
NSt0	221million	219million	188million	85.82	28.98	98.98
NSt1	192million	190million	164million	86.39	25.35	98.26
NSt2	200million	199million	170million	85.46	26.16	99.18
NFt0	223million	221million	193million	86.96	29.66	99.33
NFt1	244million	243million	216million	88.92	33.22	99.1
NFt2	289million	288million	250million	86.87	38.56	99.9

**Fig. 1:**
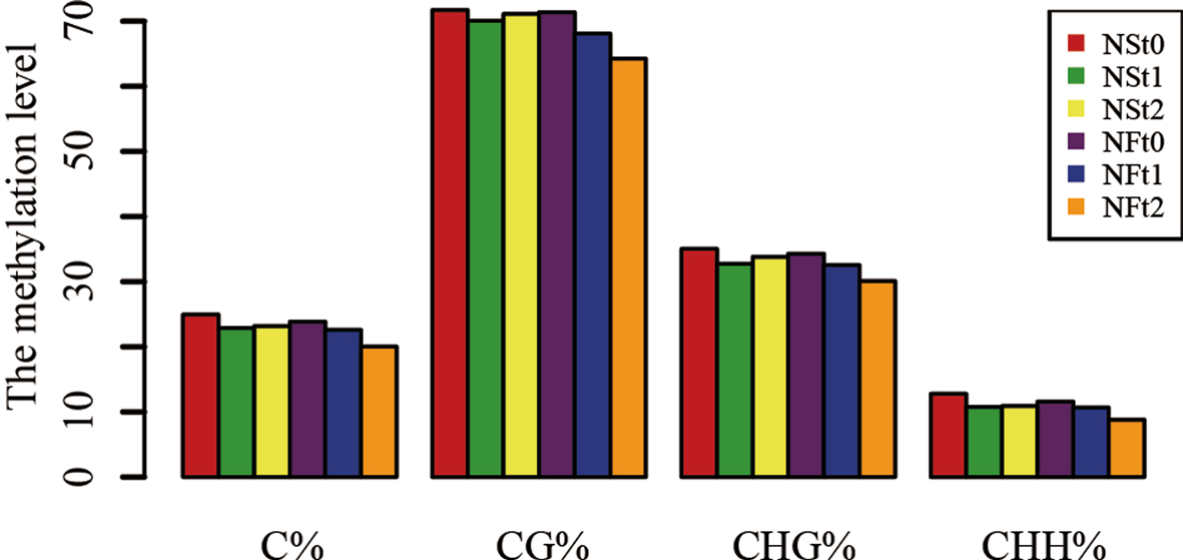
The average DNA methylation leves of two rapeseed cultivars in genome-wide methylated cytosine(C), and in the CG,CHG and CHH contexts

**Fig. 2:**
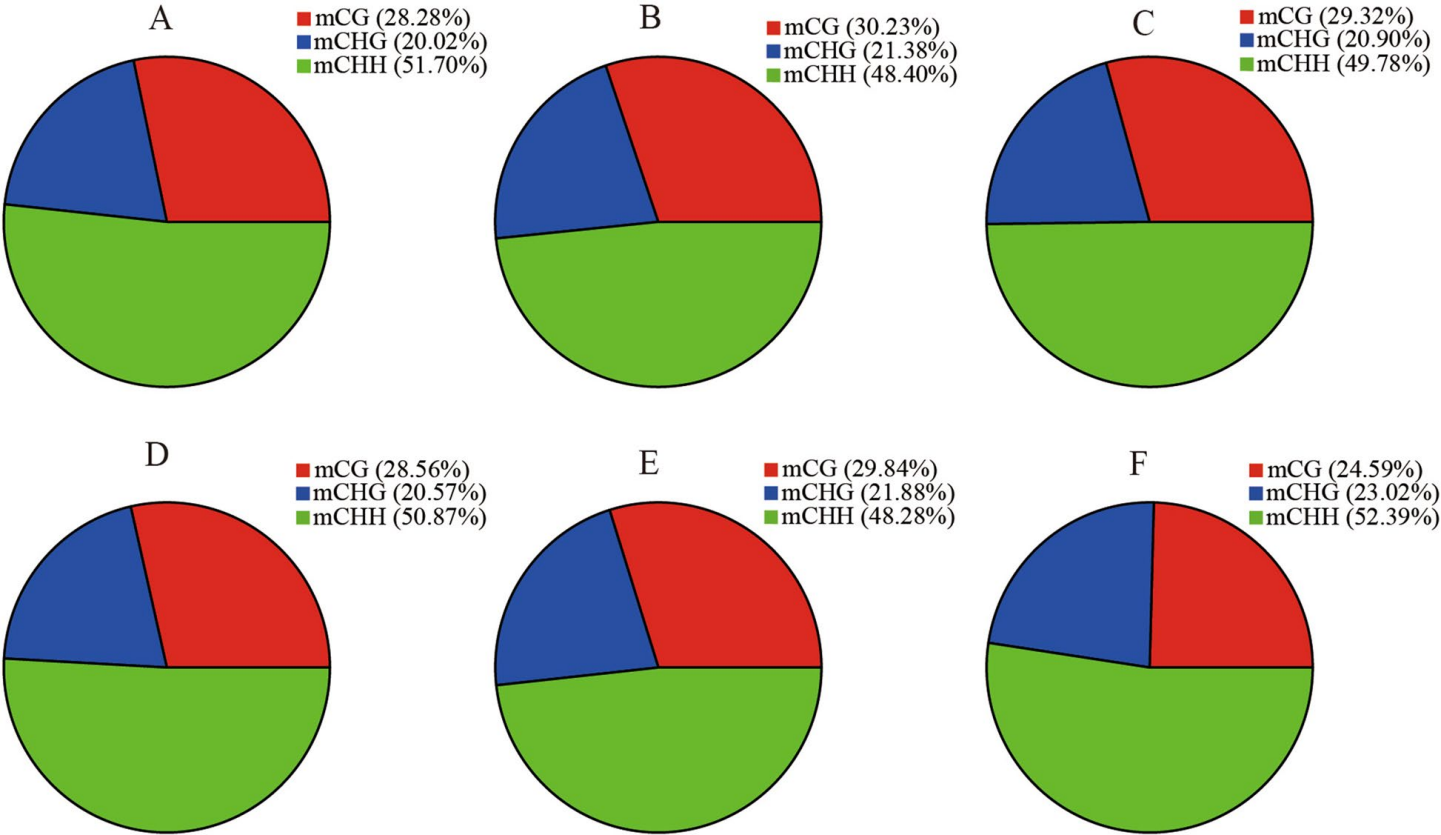
The percentages of methylated cytosines under CG, CHG, and CHH contexts. **A** the percentages of methylated cytosines in NSt0; **B** the percentages of methylated cytosines in NSt1; **C** the percentages of methylated cytosines in NSt2; **D** the percentages of methylated cytosines in NFt0; **E** the percentages of methylated cytosines in NFt1; **F** the percentages of methylated cytosines in NFt2

The average methylation level showed a downward trend in winter rapeseed after freezing stress. The trend was similar in CG, CHG, CHH sequence sites(where H = A, T, or C) and more pronounced in NF than NS (Fig. 1). However, comparing the two cultivars showed that methylation levels were hardly different before freezing treatment, but the methylation level of NF was lower than that of NS after freezing treatment, especially in the t_2_ treatment. It is worth noting that among three sequence contexts, CG has the highest methylation level, followed by CHG and CHH. Furthermore, almost half of 5-methylcytosines (5-mCs) occurred at CHH sequence site in all samples, with a lower proportion occurring at CG and CHG sites (Fig. 2).

**Fig. 3:**
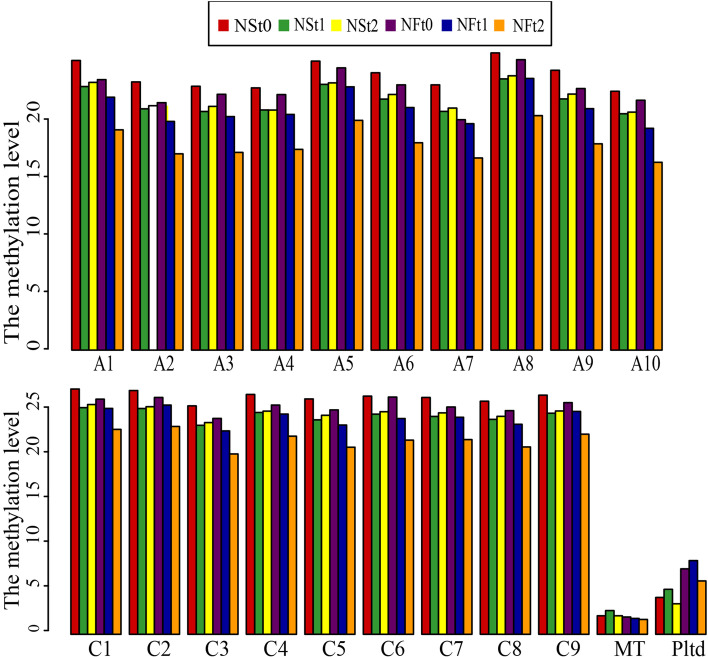
Distribution of methylation levels in different chromosemes of two rapeseed cultivars

The methylation levels of all 19 chromosomes in *B.napus* leaves showed that the C sub-genomes methylation level was higher than the A sub-genomes (Fig. 3). After freezing stress, all 19 rapeseed chromosomes showed a downward methylation level trend (Fig. 3; Supplementary Table S1). Interestingly, the average methylation levels of mitochondrion (mt) and chloroplast (cp) DNA were lower than in *B.napus* nuclear chromosomes; however, the methylation levels of mitDNA and cpDNA increased with treatment. At the same time, the chloroplastt's methylation level was higher than the mitochondrion's under the same conditions.

**Fig. 4:**
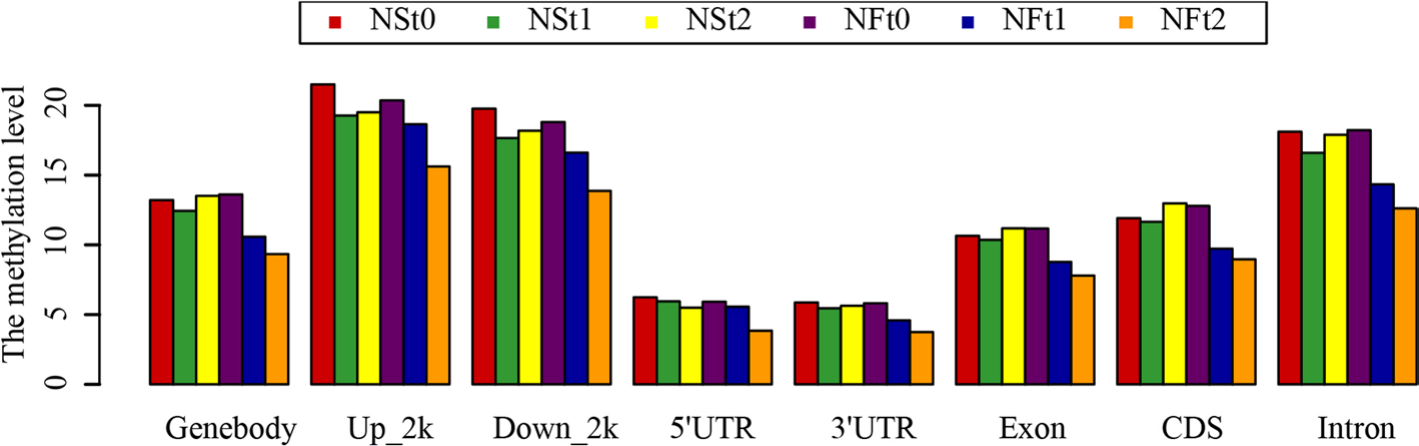
Distribution of methylation levels in different genomic functional regions of two rapeseed cultivars

The methylation patterns among gene features in response to freezing stress were compared in different genome regions of six libraries to further identify the methylation patterns. Eight functional regions of the genome, gene body, exons, introns, protein-coding sequences (CDS), 5' untranslated regions (5'UTR), 3' untranslated region (3’UTR), 2 kb upstream, and 2 kb downstream, showed a decline in methylation after freezing stress (Fig. 4). Similarly, the CHH sites showed the lowest methylation levels, while CG had the highest of these functional regions (Supplementary Figure S1). A comparison of different functional regions demonstrated that the methylation levels were highest in the 2 kb upstream and 2 kb downstream, followed by an introns, with the lower the methylation levels in gene body, CDS, Exons, 5'UTR, 3'UTR region. Based on these results, it is speculated that methylation may play a role in the regulatory region of genes to activate gene expression.

**Fig. 5:**
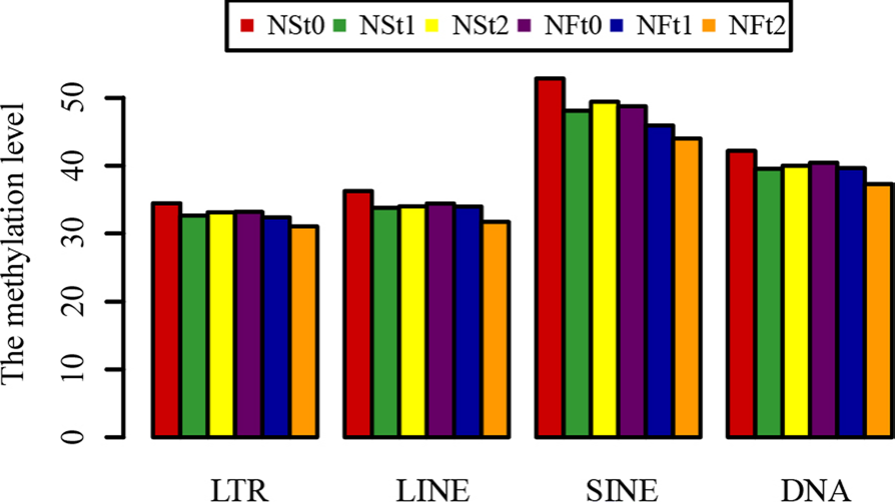
Distribution of methylation levels in different transposable elements of two rapeseed cultivars

Because different transposable elements (TEs) have different functions, the methylation levels between different TEs can affect their function. Therefore, three types of TEs, long terminal repeats (LTRs), long interspersed nuclear elements (LINEs), short interspersed nuclear elements (SINEs), and DNA methylation levels were analyzed. The result showed that the SINEs had the highest methylation level among the rapeseed TEs in the rapeseed genome, and SINEs had the highest methylation levels in the CHG and CHHsequences but had the lowest methylation level in the CG context (Fig. 5; Supplementary Figure S2). All TEs types in all samples had a similar downward trend after freezing stress, which was closely related to the activation of more stress genes' under freezing stress.

**Fig. 6:**
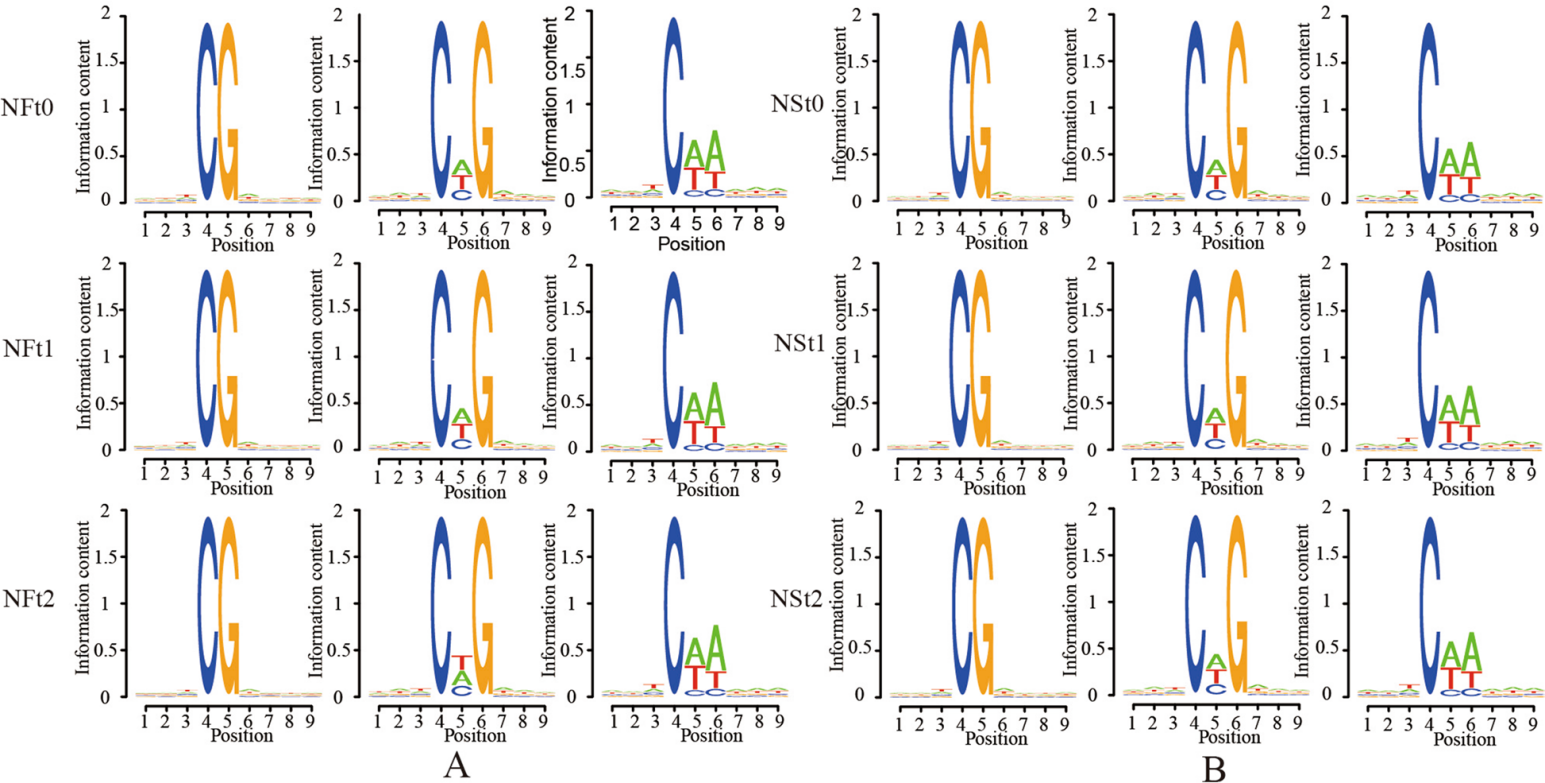
Sequence preference round all methylated cytosine of rapeseed genome under CG, CHG and CHH contexts. **A** The sequence preference of NF in t0, t1, and t2 treatments under CG, CHG and CHH contexts. **B** The sequence preference of NS in t0, t1, and t2 treatments under CG, CHG and CHH contexts

Because DNA methylation sites were highly correlated with DNA sequence, the 9 bp sequence information near the methylation sites in three contexts was analyzed to obtain the sequence preference around all mCs in the rapeseed genome. The result suggested that the methylation always occurred at the TCGA sequence in the CG sites (Fig. 6). Moreover, CTG and CAA sequences were the most common sequence motifs at the CHG and CHH sites. Unexpectedly, the sequence preference of cytosine DNA methylation did not differ between two cultivars and between freezing stress and control, and TCGA, CTG, and CAA sequencees dominated in all samples under CG, CHG, and CHH contexts, indicating that the new cytosine preferentially occurs in the above dominate sequences, and the sequence preference of cytosine DNA methylation sits in the rapeseed genome did not alter under freezing stress.

### Analysis of differentially methylated regions (DMRs) and differentially methylated genes (DMGs) between two cultivars

**Fig. 7:**
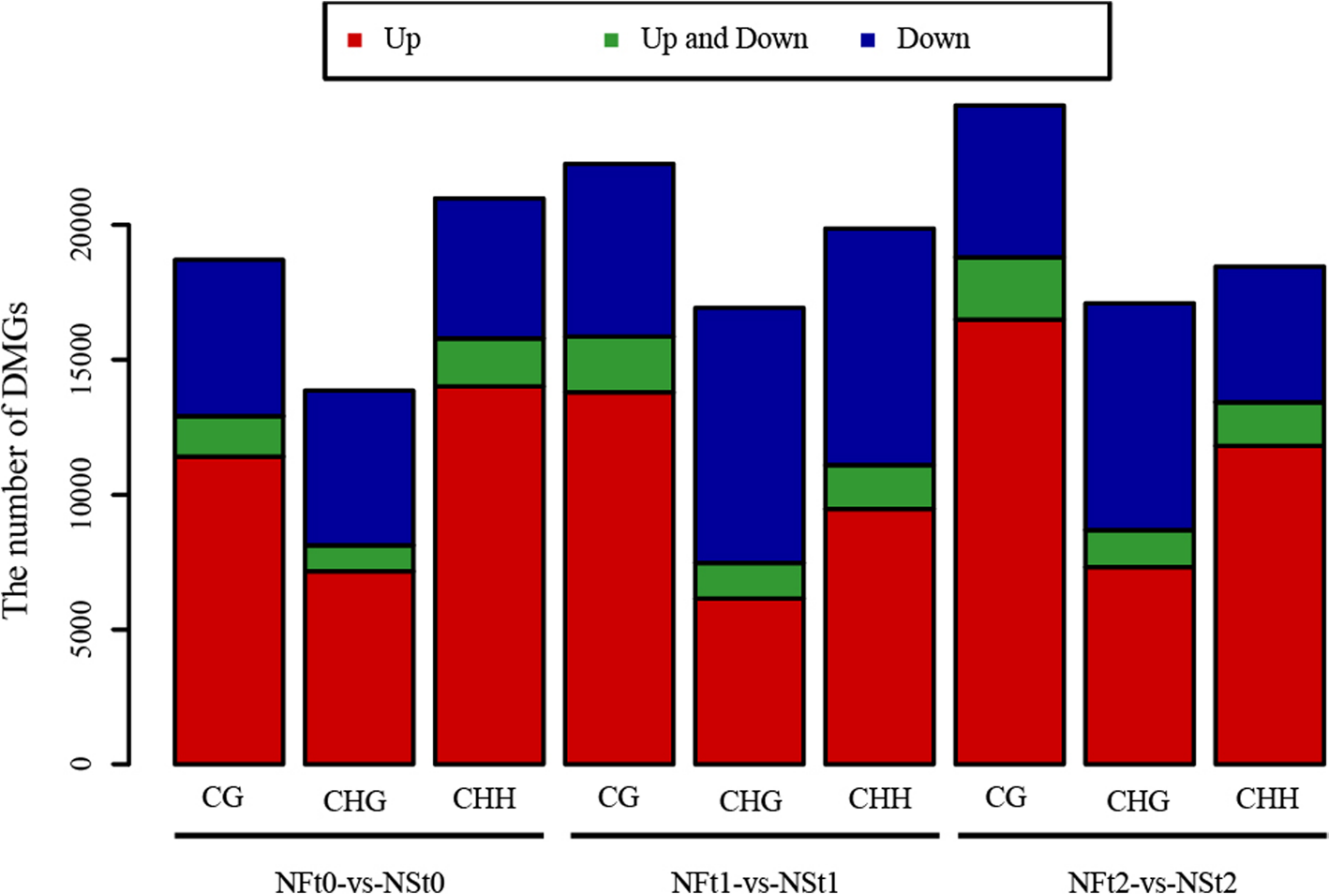
Number of diferentially methylated genes identified between two rapeseed cultivars under CG, CHG and CHH contexts

To understand the difference in DNA methylation in two cultivars with varying tolerance to freezing stress, we compared the DMRs of of NF vs. NS at different treatments at CG, CHG, and CHH sites. In the CG context, 37,898, 47,952, and 60,246 DMRs showed hypermethylation, and 21,258, 25,915, and 23,734 DMRs showed hypomethylation in NFt0 vs. NSt0, NFt1 vs. NSt1, and NFt2 vs. NSt2 respectively (Supplementary Table.S1, S2). For the CHG context, 58,354, 88,277, and 86,366 DMRs were identified in the groups of NFt0 vs. NSt0, NFt1 vs. NSt1, and NFt2 vs. NSt2, including 32,014, 28,595, and 35,333 hypermethylated DMRs and 26,340, 59,682, and 51,033 hypomethylated DMRs, respectively. Similarly, 71,983 (54,417 hyper and 17,566 hypo), 59,691 (29,182 hyper and 30,509 hypos), and 58,658 (42,665 hyper and 15,993 hypos) DMRs were identified in NFt0 vs. NSt0, NFt1 vs. NSt1, and NFt2 vs. NSt2 under CHH context, respectively. Genes overlapping with DMRs for at least 1 bp in the functional region were defined as DMGs. In total of 18,698, 13,847, and 20,981 DMGs were identified in NFt0 vs. NSt0 under the CG, CHG, and CHH context (Fig. 7; Supplementary Table S3). In NFt1 vs. NSt1, 22,261, 16,915, and 19,864 DMGs were obtained in the CG, CHG, and CHH context. Similarly, under three contexts, 24,426,17,088, and 18,452 DMGs were identified in NFt2 vs. NSt2. The number of hypermethylated genes in CG and CHG increased after freezing stress treatment (t1 and t2) compared to the control (t0). However, the number of hypermethylated genes decreased in the CHH context, indicating that hypermethylation mainly occurred in CG and CHG sites during freezing stress.

### RNA sequencing analysis and defining differentially expressed genes (DEGs) between two cultivars

**Fig. 8:**
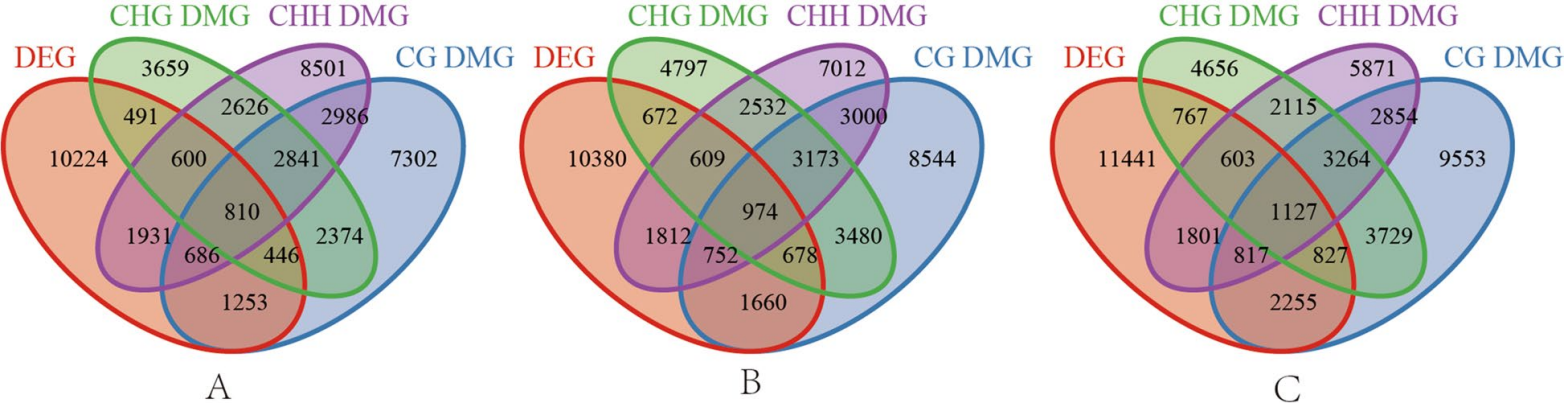
Number of DMEGs identified between two rapeseed cultivars. **A** The number of DMEGs in NFt0 vs. NSt0; **B** the number of DMEGs in NFt1 vs. NSt1; **C** the number of DMEGs in NFt2 vs. NSt2

RNA sequencing (ribonucleic acid -seq) was also performed on eighteen libraries from six samples (three biological replicates for per sample) of two cultivars to investigate the association between DNA methylation and gene expression. The high-quality clean reads per library were more than 39 million, the Q30 for all libraries was greater than 94%, and the GC content per library was approximately 48%(Supplementary Table S4). For all libraries, over 77% of reads, were uniquely mapped to the reference genome and the mapping ratio was close to 80%. The correlation analysis showed that the correlation coefficient between the three replicates of any particular sample was extremely high, indicating that the experiment was reliable and reasonable(Supplementary Table S4). A total of 16,441 DEGs were identified in the NFt0 vs NSt0 sample, which including 9204 up-regulated genes and 7237 down-regulated genes(Fig. 8; Supplementary Table S5). Compared to controls, 17,537 (up 11,345 and down 6192) and 19,638 (up 10,222 and down 9416) DEGs were identified in the NFt1 vs. NSt1 and NFt2 vs. NSt2 groups(Fig. 8; Supplementary Table S5). The number of DEGs increased after freezing treatment, indicating that some genes were aroused during freezing stress.

### Comparative analysis of DEGs associated with DMGs between two cultivars

**Fig. 9:**
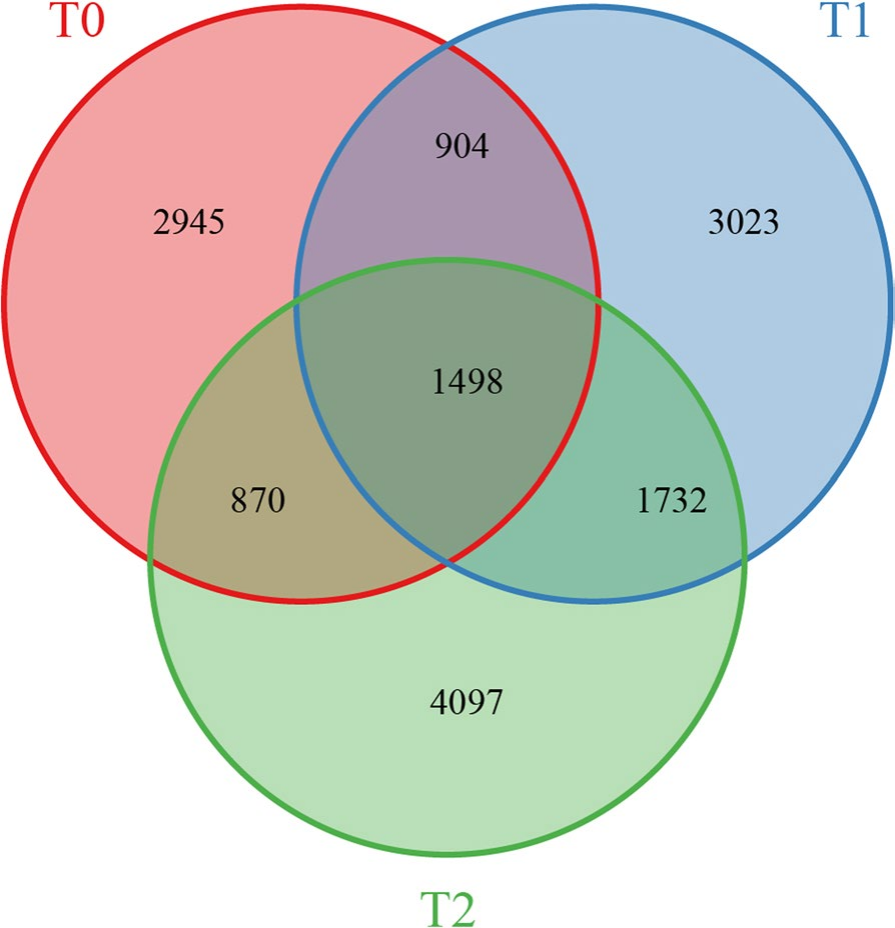
Number of DMEGs identified between two rapeseed cultivars at three treatments

To further explore the possible association between DNA methylation and gene expression, firstly, the overlap between the DMGs and DEGs of two cultivars was examined at CG, CHG, and CHH sites. In the NFt0 vs. NSt0 of 3195, 2347, and 4027 DMEGs were identified in under CG, CHG, and CHH contexts (Fig. 8; Supplementary S6). After merging DMEGs from CG, CHG, and CHH context, 6217 DMEGs were obtained in the NFt0 vs. NSt0 (Fig. 8). After freezing stress for 12 h, 4064, 2933, and 4147 DMEGs were determined in the NFt1 vs. NSt1 sample at the CG, CHG, and CHH sites, and 7157 DMEGs were obtained after combination at CG, CHG, and CHH contexts (Fig. 8; Supplementary S6). After 24 h of freezing stress, 5026, 3324, and 4348 DMEGs were identified in the NFt2 vs. NSt2 under the CG, CHG, and CHH contexts, and 8197 DMEGs were obtained after combination at CG, CHG, and CHH contexts(Fig. 8; Supplementary S6). The DMEGs of NFt0 vs. NSt0 were removed from the overlapping DMEGs of NFt1 vs. NSt1 and NFt2 vs. NSt2 to obtain 1732 DMEGs, which will be used for subsequent functional analysis (Fig. 9).

### GO annotation and KEGG pathway enrichment analysis of DMEGs

**Fig. 10:**
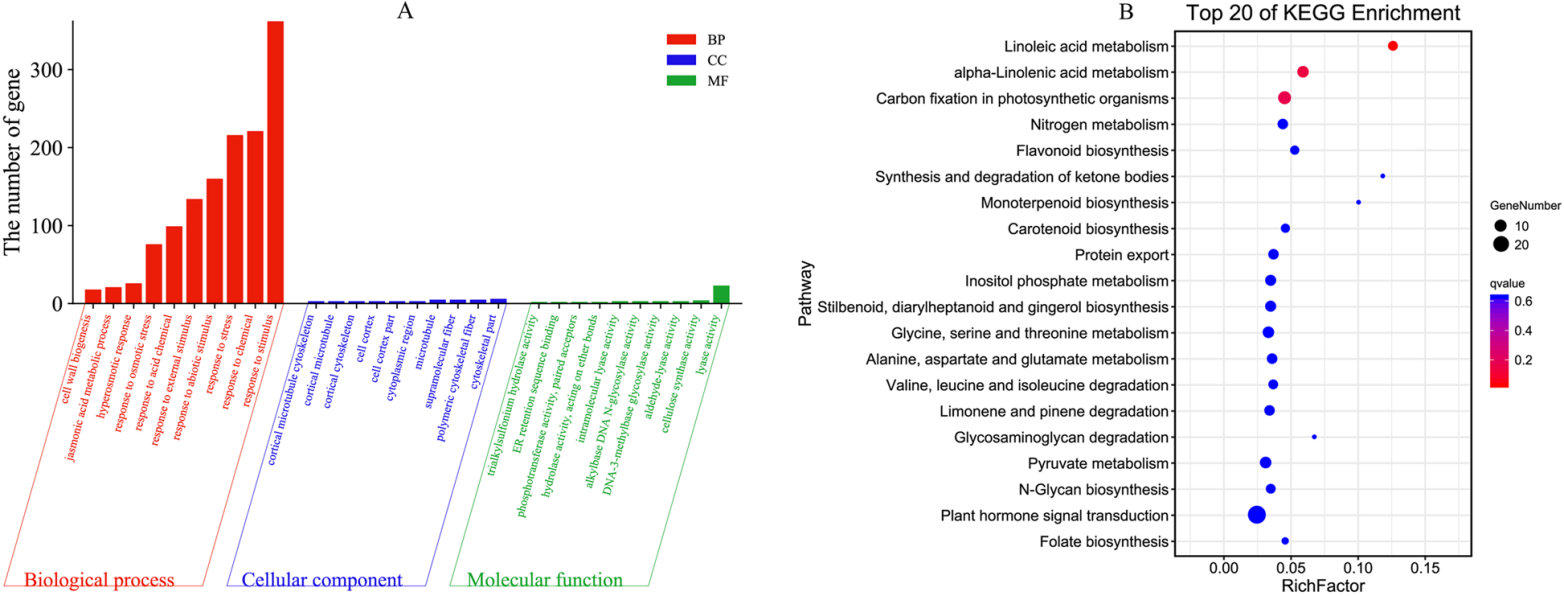
The functional annotation of DMEGs. A GO annotation of DMEGs; B KEGG pathway enrichment of DMEGs

To understand the potential role of DNA methylation patterns in freezing tolerance, 1732 DMEGs were subjected to gene ontology (GO) classification annotation (Fig. 10A; Supplementary Table S7). Those DMEGs were annotated to 1647 functional categories, including 1143 biological processes, 161 cellular components, and 343 molecular functions. They were found to be mainly involved in biological processes, such as response to external stimulus, response to stimulus, response to osmotic stress, response to abiotic stimulus, and response to stress. In addition, cytoskeletal part, supramolecular fiber, and microtubule were the terms that significantly dominated the cellular components. The endoplasmic reticulum (ER) retention sequence binding, phosphotransferase activity, DNA-3-methyl bases glycosylase activity, and C-methyltransferase activity were the most represented GO categories in the molecular functions.

The 1732 DMEGs identified in winter rapeseed were submitted to KEGG pathway enrichment analysis to determine their complex biological functions (Fig. 10B; Supplementary Table S7). A total of 343 DMEGs were mapped successfully to 113 KEGG pathways. The top 20 KEGG enrichment pathways are shown that linoleic acid metabolism, alpha-linolenic acid metabolism, carbon fixation in photosynthetic organisms, and flavonoid biosynthesis were significantly enriched. Besides, plant hormone signal transduction and some of the amino acid metabolism pathways were also enriched.

### Identification of differentially expressed transcription factors for DMEGs

**Fig. 11:**
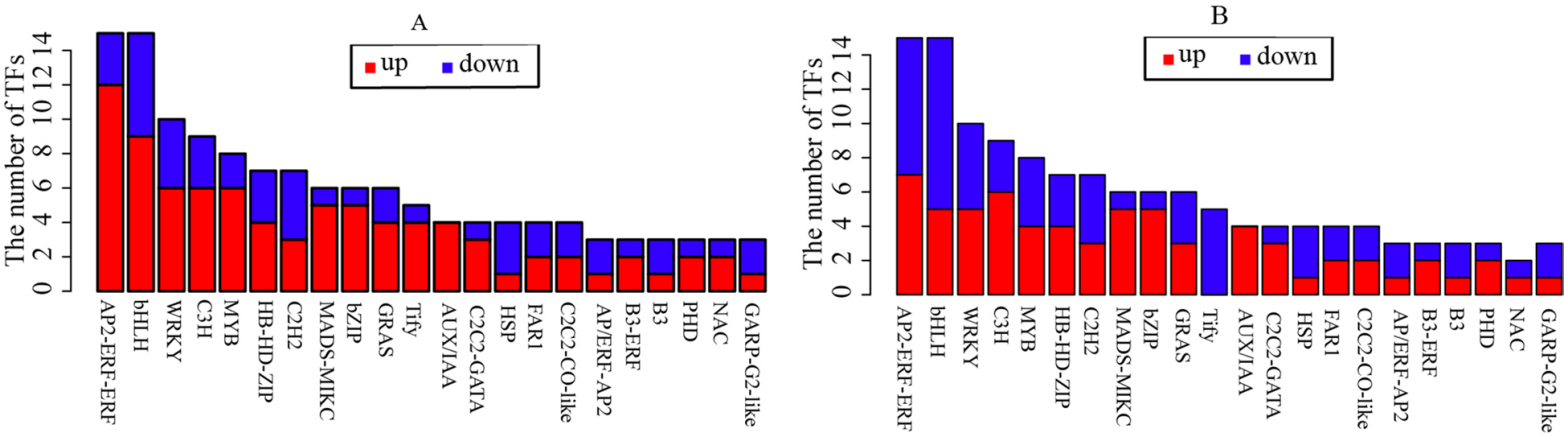
Classification of differentially expressed TFs between two winter rapeseed under freezing treatment. **A** The unmber of differentially expressed TFs in NSt1 vs. NFt1; **B** the unmber of differentially expressed TFs in NSt2 vs. NFt2

One-hundred and eighty-three 183 transcription factors (TFs) were identified from 1732 overlapped DMEGs in NFt1 vs. NSt1 and NFt2 vs. NSt2 groups.Most TFs were up-regulated, including ethylene response factor (AP2/ERF), transcription factor bHLH (bHLH), WRKY transcription factor (WRKY), zinc finger CCCH domain-containing protein (C3H), transcription factor MYB (MYB), basic leucine zipper (bZIP), homeobox-leucine zipper protein (HB), protein TIFY (TIFY), auxin-responsive protein (AUX/IAA), and GATA transcription factor (C2C2-GATA) families. Most of the down-regulated TFs belonged to the auxin response factor (B3-ARF), heat stress transcription factor (HSF), zinc finger protein (C2H2) (Fig. 11; Supplementary Table S8). Correlation analysis showed that the overall methylation level was significantly negatively correlated with the gene expression level (*p* < 0.05), but the correlation between different sites and gene regions was inconsistent (Supplementary Table S8). These results suggested that methylation played an important role in the process of activating TFs under freezing stress, especially the up-regulated TFs, thereby regulating the expression of downstream genes in response to freezing stress.

### RNA-Seq validation of DMEGs by quantitative Real-Time PCR

**Fig. 12:**
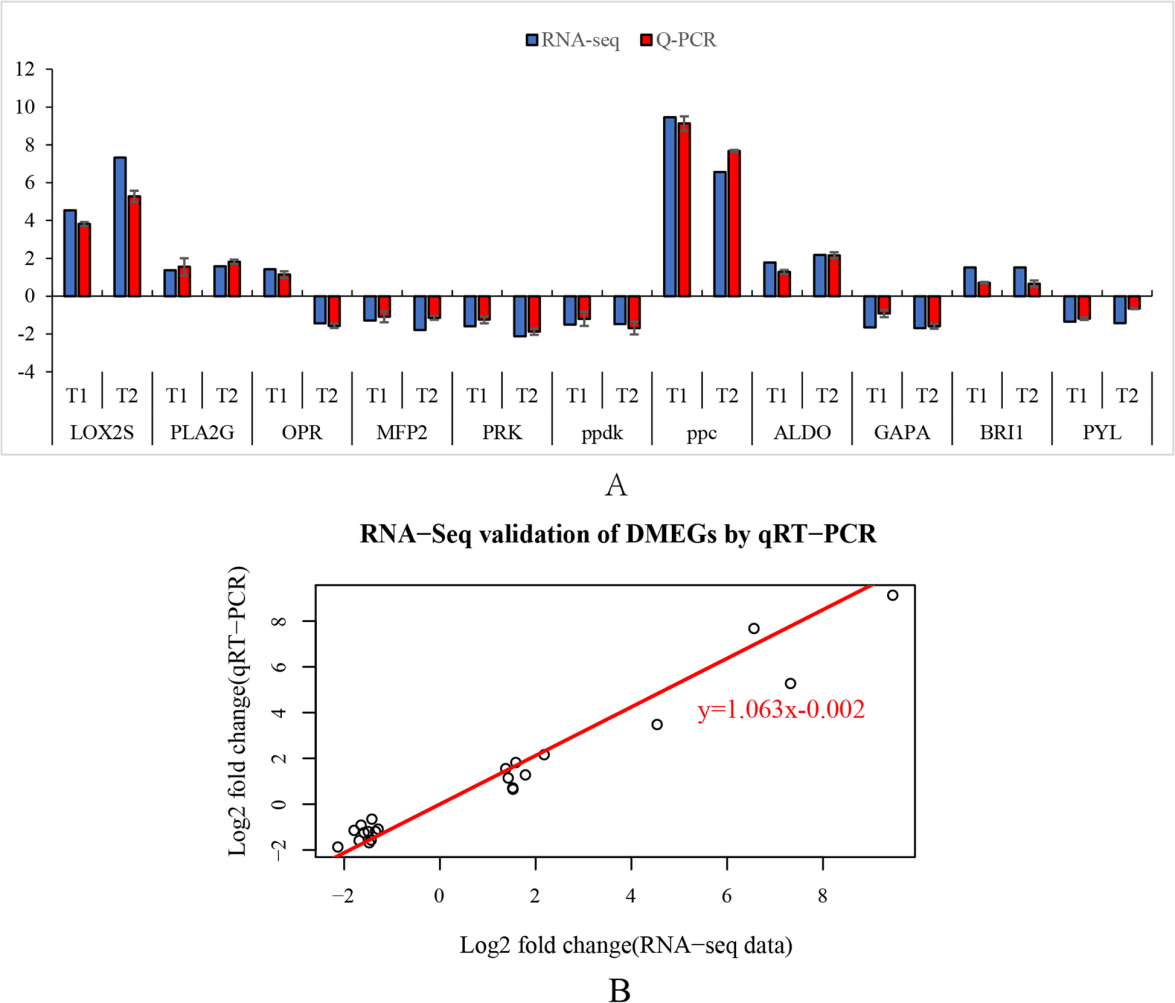
Comparative analysis of mRNA and RNA-seq levels between two rapeseed cultivars under freezing stress. **A** The column chart between mRNA and RNA-seq level; **B** Concordance changes between mRNA and RNA-seq levels. The data is the log_2_ value of the ratio of NS gene expression value to NF expression value under freezing treatment (t1 and t2).Values are means ± SD (from three biological replicates) of Q-PCR data under freezing treatment

Based on KEGG enrichment analysis, eleven genes related to plant hormone signal transduction and linoleic acid metabolism pathways in rapeseed under freezing stress were selected to validate the reliability of RNA-seq data by quantitative Real-Time PCR (qRT-PCR). Most genes were found to be changed consistently at both the qRT-PCR and RNA-seq data under freezing stress in rapeseed cultivar NS compared to cultivar NF, despite differences in expression levels of some genes. (Fig. 12; Supplementary Table S9). These results suggested that the RNA-seq results were true and reliable, which can be used in the present study.

## Discussion

**Fig. 13:**
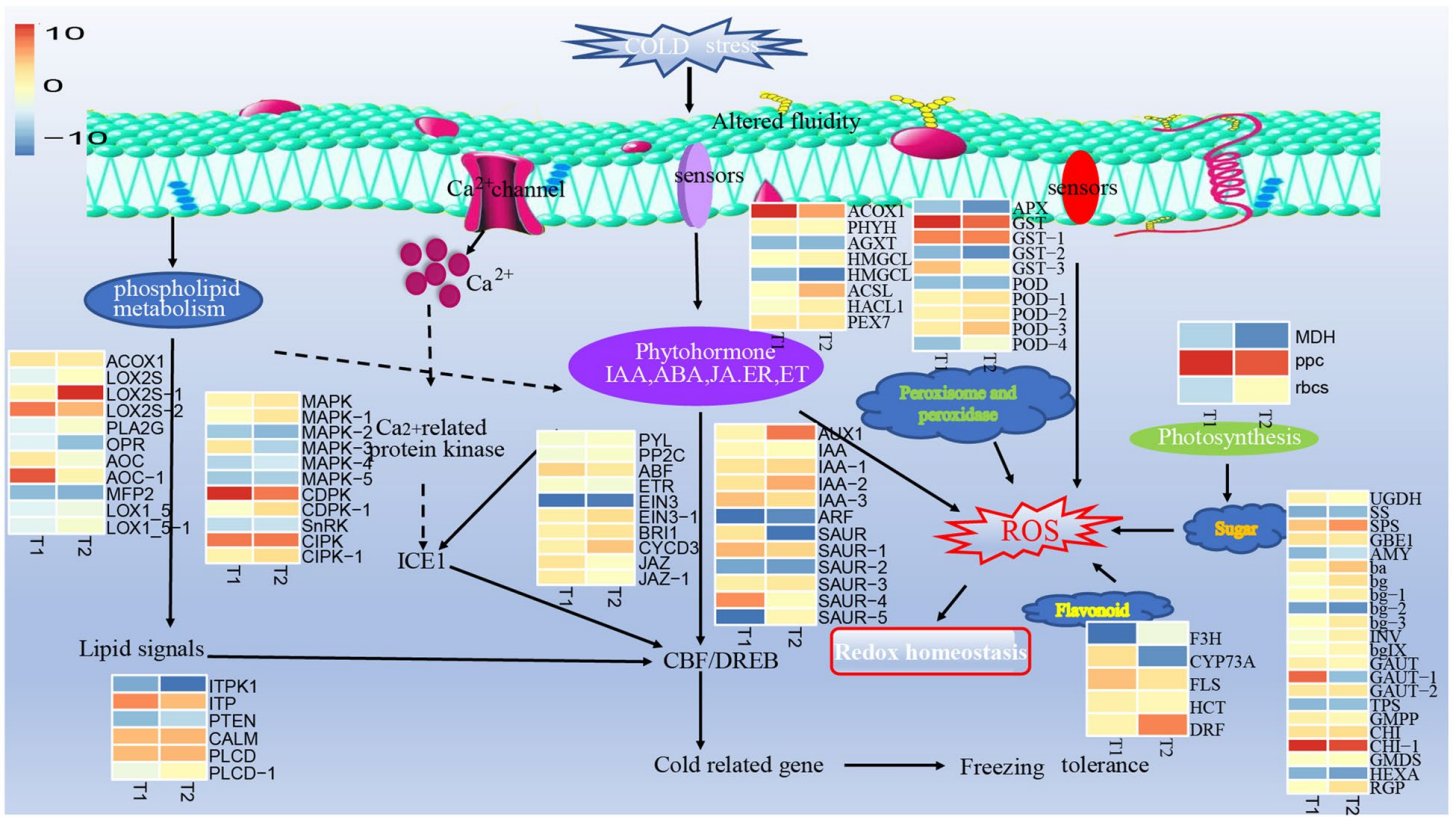
A possible model of the freezing tolerance mechanism regulated by methylation in winter rapeseed. The solid line arrow represent metabolic pathways and cellular processes. Dashed arrows indicate possible regulation. Heat maps indicate the log2 value of the ratio of NS gene expression value to NF expression value under freezing treatment (t1 and t2)

Rapeseed is China's primary oilseed crop, including, including the winter rapeseed (over 90%) and little of spring rapeseed [17]. Previous research indicated that the 35° north latitude (Tianshui in the Gansu Province) was the northernmost limit of *B. napus* winter rapeseed of in China [18]. The cultivated spring rapeseed and B. rapa can survive north of 35°N [19, 20]. Over the past decade, our research group has successfully selected and bred rapeseed cultivars of B. napus that can overwinter safely in most of the northwestern areas from the 38° to the 42° north latitude, where the lowest temperature in winter is -26℃ [15, 16]. Moreover, the cold tolerance characteristics of these rapeseed cultivars at the transcription, protein, physiological, biochemical, and subcellular levels were studied [15, 21, 22]. The strong cold-resistant cultivar had stronger antioxidant enzyme activity and accumulation of cryoprotective molecules, associated with the metabolic pathways, including alpha-linolenic acid metabolism, plant hormone signal transduction, microbial metabolism in diverse environments, linoleic acid metabolism, phenylpropanoid biosynthesis, glutathione metabolism, flavonoid biosynthesis metabolism, and amino acid metabolism. DNA methylation, the main epigenetic modification, was involved in regulating plant adaptation to abiotic stresses and was inherited by offspring [8, 9, 23]. However, the current understanding of the methylation pattern amid freezing stress and the complex mechanisms of methylation regulating freezing tolerance in winter rapeseed is still limited. Therefore, in the current study, the whole-genome bisulfite sequencing (WGBS) was used to analyze the methylation pattern of NS and NF cultivars under freezing stress. The integrative analysis of methylome and transcriptome will pave the pathway to understanding the cold tolerance mechanism of winter rapeseed (Fig. 13).

### Methylation level changes of winter rapeseed under freezing stress

Enzymes regulate methylation and demethylation in plants, which are the part of stress reactions to respond to environmental conditions [24, 25]. It is proposed that DNA methylation is related to the stress memory of plants so that their offspring can respond more quickly and accurately to the same stress [7]. This is consistent with our theory of enhancing the cold resistance of rapeseed through cold acclimation, and it also exists in other crops. Current methods have been to detect the methylation dynamics, such as methylation-sensitive amplification polymorphism (MSAP), methylation-specific PCR (MSP), and WGBS. The WGBS method is widely used in plant methylation research due to its high accuracy, wide range, high reliability, and cost-effectiveness [26–, 27–29]. In winter rapeseed, previous studies using the MSAP method had shown that the methylation level decreased after low-temperature stress. In this study, the methylation levels of the two cultivars showed a downward trend in all contexts following the freezing stress, and the effect of freezing-sensitive cultivars was stronger than freezing-resistance cultivars. Notably, the methylation level of CG was highest among the three contexts, while the proportion of CHH was abundant, which was reported by other [30]. Previous studies revealed that the DNA methylation level in the CG context was positively correlated with plants' genome size, 30.5% in *Arabidopsis* and 92.5% in Beta vulgaris [31, 32]. In this study, we found that the methylation level in leaves of Brassica napus was 64.24–71.70% under the CG context (Fig. 1). The methylation level is also related to the tissue parts of the plant [30]. Examination of rapeseed's 19 chromosomes indicated that the C sub-genomes methylation level was higher than in A sub-genomes (Fig. 3). A similar result was obtained in the Genic Male Sterile Line, the Restorer Line, and cultured microspores of rapeseed [30, 33]. Furthermore, we found that the methylation levels of cpDNA and mtDNA were much lower than those of nuclear, and the mtDNA methylation levels were lower than that of cpDNA. These results are consistent with previous report on nuclear DNA, cpDNA and mtDNA methylation [34]. In addition, their research indicated the methylation level first increased and then decreased under freezing stress, which further suggest that the methylation role of chloroplast and mitochondrial DNA control gene expression in plant response to abiotic stress. The comparison between different functional regions depicted that the methylation level was highest in the 2 kb upstream and 2 kb downstream regions, followed by intron, while that in gene body, CDS, exons, 5'UTR, and 3'UTR regions were lower, indicating that the methylation plays a role in the genes' regulatory region (Fig. 4). Simultaneously, the SINEs had the highest average methylation levels, and LTRs had the lowest methylation level among TEs (Fig. 5). Furthermore, the prefered sequence of DNA methylation for winter rapeseed did not change after freezing stress (Fig. 6). In the integrated analysis between DMGs and DEGs, 1732 DMEGs with significant change were yielded under the t1 and t2 treatments for the freezing-resistant (NS) cultivar relative to freezing-sensitive (NF) cultivar (Fig. 8– 9). Function enrichment analysis showed that DMEGs were mainly involved in linoleic acid metabolism, alpha-linolenic acid metabolism, carbon fixation in photosynthetic organisms, nitrogen metabolism, flavonoid biosynthesis, plant hormone signal transduction, and some amino acid metabolism pathways (Fig. 10; Supplementary Table S7).

### Methylation-regulated transcription factors and protein kinases

Many transcription factors have been reported participate to convey cold stress signals. Among them, the C-repeat binding transcription factor (CBF) is a central player in freezing tolerance. The inducer of C-repeat-binding factor expression 1 (ICE1) activate CBF and with it the expression of downstream cold-regulated genes, thereby enhancing freezing tolerance [35, 36]. The ICE1 belongs to the transcription factor bHLH family, and it positively regulates the CBF expression to contribute to freezing stress. In addition, many transcription factors have been determined to positively participate in the cold resistance of plants, including AP2/ERF, MYB88 /MYB124, WRKY, bZIP, C3H, and TIFY [15–, 37–41]. In this study, most AP2/ERF, WRKY, MYB, bZIP, C3H, TIFY, AUX, and HB transcription factors were up-regulated in cultivar NS compared to cultivar NF, but some member of TFs were down-regulated(Fig. 12; Supplementary Table S8). Hypermethylation of gene body in CG, CHG, and CHH contexts caused up- or downregulated expression of transcription factors under Phosphate Starvation Stress in Rice [42]. Also, the overall methylation level was significantly negatively correlated with the gene expression level (*p* < 0.05), but the correlation of different loci and gene positions was different, and the methylation level was also positively correlated with the expression level.(Supplementary Table S8). We find that some members of up-regulated TFs, such as MYBs, WRKY, AP2/ERF, TIFY, and bZIPs, were hypomethylated in cultivar NS compared to cultivar NF in different gene regions, while the some members of up-regulated TFs were hypermethylated. Meanwhile, most of TFs have different methylation level in different gene regions and different contexts, so the methylation effect of TFs also need further research in future works. It has been reported that several protein kinases regulated the cold stress response of plants by a cold-sensing calcium channel [43]. The mitogen-activated protein kinases (MAPKs), Ca^2+^-dependent protein kinases (CDPKs), calcium/calmodulin-regulated receptor-like kinases (CRLKs), calcineurin-B-like interacting protein kinases (CIPKs), dehydration-responsive element-binding protein kinases (DREB), and receptor-like kinases (RLKs) have also been identified as key protein in the Ca^2+^ signaling pathway [43–45]. Similar results were obtained in this study; more than two-thirds of protein kinases were up-regulated in NS compared to NF after freezing stress (Fig. 13; Supplementary Table S8). The result of methylation showed the methylation regulation patterns of homologous genes or genes' different regions were inconsistent. These results showed that the protein kinases and TFs might be activated by the Ca^2+^ signaling in rapeseed after cold treatment, and triggering the expressions of downstream freezing responsive genes; the methylation might regulate the Ca^2+^ signaling by adjusting protein kinases and TFs.

### Methylation-regulated lipid metabolism

Under cold stress, the plant cell membrane alters the fatty acid profile and loses fluidity; however, increasing the unsaturation in the membrane lipids can maintain the normal fluidity of membranes and enhance the tolerance to freezing stress [46, 47]. The secretory phospholipase A2 (PLA2G), a key enzyme that converts phosphatidylcholine into linoleic acid and linolenic acid, could alter the lipid composition and improve the membrane lipid unsaturation. In the present study, the expression of *PLA2G* in NS was higher than that in NF after freezing stress, and it was hypomethylated in the 2 kb downstream region (Fig. 13; Supplementary Table S10). Plant lipoxygenases are a kind of fatty acid dioxygenases with diverse functions, which not only participate in the peroxidation of linolenic and linoleic acids, and start the synthesis of jasmonic acid [48]. It was reported that jasmonic acid (JA) positively modulates the CBF pathway, leading to regulating stomatal closure and maintaining photosynthesis under cold stress by accumulating cryoprotective compounds and interacting with other plant phytohormones [49, 50]. In the study, eight DMEGs, including three lipoxygenase (LOX2S), two allene oxide cyclase (AOC), one acyl-CoA oxidase (AOCX), one 12-oxophytodienoic acid reductase(OPR), and one enoyl-CoA hydratase/3-hydroxyacyl-CoA dehydrogenase (MFP2), involved in linoleic acid, alpha-linolenic acid metabolism and the JA synthesis, and all genes were up-regulated in NS compared to NF after freezing stress. However, 2 kb the downstream region methylation levels were down-regulated (Fig. 13; Supplementary Table S10).In addition, lipid metabolism generates the lipid signal that activates the downstream cold-responsive genes [51]. This study showed that six genes involved the lipid signal transmission, half the number of genes were up-regulated in NS compared to NF after freezing stress, and it was hypermethylated (Fig. 13; Supplementary Table S10). Our results demonstrated that the fatty acid β-oxidation and JA biosynthesis played a central role in plant freezing tolerance, and DNA methylation regulated the expression of the related gene.

### Methylation-regulated carbon fixation and sugar metabolism

Photosynthesis is a primary plant energy source and maintains the normal life activities of cells [52]. The fixation capacity of CO2 can reflect the photosynthetic rate of plants. Photosynthesis was easily affected under cold stress, which leads to excessive energy generation and even triggers photoinhibition [53]. The ribulose-bisphosphate carboxylase (RBCs) is the elite enzyme of the Calvin cycle, which converts airborne CO2 into sugars. In addition, the first reaction of the gluconeogenesis pathway can also fix CO2 to sugar, phosphoenolpyruvate carboxylase (PPC) is the core enzyme in this pathway, and phosphoenolpyruvate carboxykinase (PCA) has the opposite function to supplementary amount of pyruvate. The research indicated that the RBCs and PPC were up-regulated, the PCA was down-regulated in NS compared to NF, and they were hypermethylated in 2 kb upstream position (Fig. 13; Supplementary Table S10). The paper's research indicated that the soluble sugars play an important role in maintaining a cellular osmotic balance of plants under abiotic stress [54, 55]. Soluble sugars mainly include monosaccharides and oligosaccharides, which arose from photosynthetic fixation and hydrolysis of polysaccharides and glycoconjugate. The study indicated that many genes encoding amino sugar, nucleotide sugar, starch, and sucrose metabolism, most of the genes were up-regulated expression after freezing stress and were hypermethylated (Fig. 13; Supplementary Table S10). The beta-fructofuranosidase (INV) is a core enzyme from sucrose/sucrose-6P to β-D-fructose or α-D-glucose 6-phosphate, and the α-D-glucose 6-phosphate could enter another metabolism, facilitating the conversion of polysaccharides into soluble sugars. Simultaneously, beta-glucosidase (bgIX) and beta-amylase (amyB), are key enzymes in the conversion of cellulose to α/β glucose or maltose [56]. Our study showed that the INV, amy B, and four out of five bgIX genes were up-regulated in NS compared to NF after freezing stress. The INV genes was hypermethylated 2 kb upstream, the amyB was hypermethylated 2 kb downstream and four genes bgIX genes were hypermethylated in 2 kb up/downstream or at the gene body.

On the other hand, some sucrose synthases (SS) and alpha-amylases (amyA) boost the synthesis of polysaccharides were down-regulated in NS compared to NF after freezing stress, and the SS was hypomethylated in NS compared to NF under t1 and was hypermethylated in t2 at the 2 kb downstream, but amyA was hypermethylated in NS compared to NF at the 2 kb downstream position after freezing stress. These results suggested that the ability of carbon fixation and polysaccharides hydrolysis of NS was higher than that of NF under freezing stress, and DNA methylation also participated in photosynthesis and sugar metabolism.

### Methylation-regulated the ROS scavenging-related metabolites

Although moderate reactive oxygen species (ROS), as the signaling role, can be involved in regulating plant stress responses and improving the ability to tolerate stress, excessive ROS can cause peroxidative injury to organisms [52, 57]. Under adverse biotic and abiotic stresses, the plants have ROS-scavenging enzymes and non-enzymatic scavengers to maintain normal cellular redox homeostasis [53]. Peroxidase and peroxisome, as important enzymes, have the function of scavenging ROS in cold stress [14]. In this study, there were ten and eight identified genes involved in the peroxidase and peroxisome pathways, respectively, and most of them were significantly enriched in NS compared to NF after freezing stress (Fig. 13; Supplementary Table S10). However, their methylation was inconsistent in different regions. In addition, flavonoids are an important member of secondary metabolites that contain various important bioactive substances, including flavonols, anthocyanidins, and some sugars; they function as an antioxidant and protect the plant from a wide range of abiotic stresses, such as heavy metal stress, ultraviolet-B, and salt stress, high-temperature damage, and other environmental stresses [54, 55]. The flavonol synthase (FLS) is an enzyme catalyzing the biosynthesis of quercetin, kaempferol, myricetin, and galanin, which were highly active non-enzymatic scavengers of ROS [58, 59]. The dihydroflavonol 4-reductase (DFR) is an enzyme for the biosynthesis of leucocyanidin, leucodelphinidin, leucopelargonidin, luteoforol, and apiforoletc, which also has an antioxidant role. In this study, two DMEGs (FLS, DFR) expressions were increased in NS compared to NF; among them the methylation levels of FLS were up-regulated under the gene body, and DFR was down-regulated under the 2 kb downstream and was up-regulated under 2 kb upstream (Fig. 13; Supplementary Table S10). The naringenin 3-dioxygenase (F3H) is a core enzyme of the flavonol biosynthesis pathway, which was up-regulated expression in NFt2 vs. NSt2, and it was hypermethylated in downstream2kb (Fig. 13; Supplementary Table S10). Before transcriptome studies, the FLS, DFR, and F3H as the key downstream gene of flavonoid biosynthesis were significantly induced under cold stress in Tetrastigma hemsleyanum [60]. Taken together, these results suggested that rapeseed cultivar NS has a stronger ability to scavenge ROS under freezing stress than the cultivar NF, and the methylation regulates the expression of the ROS scavenging-related gene.

### Methylation-regulated plant hormone signal transduction related freezing resistance

Phytohormones, small endogenous signaling molecules, interact with ROS to orchestrates the plant response to abiotic stress and drive changes in transcriptomic, metabolic, and proteomic network changes that lead to plant acclimatization and survival [49]. Previous research has shown that the plant produces large amounts of phytohormones under environmental stress, such as IAA, JA, ABA, BR, and ET, which combine endogenous substances with environmental signals to improve the defense ability against stress [61–, 62–64]. Similar results were obtained in this research; 28 DMEGs, encoding some of the responsive proteins or receptors related to hormone signals, were enriched in the plant hormone signal transduction pathway. (Fig. 13; Supplementary Table S10). Studies in *Arabidopsis* and *Rice* have shown that high levels of auxin expression can improve their frost resistance, and similar results were also obtained in the present study [61, 65]. The 12DMEGs encode auxin-responsive protein (IAA), SAUR family protein (SAUR), auxin influx carrier (AUX1), and auxin response factor (ARF). At the same time, IAA, AUX1, and two-thirds of SAUR genes were up-regulated expression, and ARFwas down-regulated expression in NS compared to NF under freezing stress(Fig. 13; Supplementary Table S10). The methylation data showed that IAA and AUX1 were hypermethylated in NFt1 vs. NSt1 and NFt2 vs. NSt2 under gene body or at the 2 kb up/downstream, and ARF was hypermethylated in NFt1 vs. NSt1 and NFt2 vs. NSt2 under CHH contexts of upstream2kb (Supplementary Table S10). JA regulates the cold tolerance of plants by inducing jasmonate ZIM domain-containing protein (JAZ), a negative regulator of the JA signaling pathway [62]. Two genes encoding JAZ showed decreased expression in the NFt2 vs. NSt2_,_ whereas methylation level increased in the 2 kb upstream and gene body regions. The abscisic acid receptor PYR/PYL family (PYL) is the receptor of abscisic acid (ABA), and protein phosphatase 2C (PP2C) is an ABA co-receptors, which forms the PYL-ABA-PP2C complex to release the SnRK2 from inhibition by the PP2C [36]. When absence of ABA, the PP2C binding SnRK2 kinases, are inactive in SnRK2 kinases [66]. Recently, it was reported that cold stress activates SnRK2.6/OST1, SnRK2.6 interacts with ICE1 and phosphorylates it to activate the CBF-COR gene-expression cascade, and improving the freezing tolerance of plants [67]. In this study, PP2C and PYL have a down-regulated expression in NS compared to NF after freezing stress, and the methylation levels were inconsistent in different regions. Therefore, we hypothesized that PP2C and PYL inhibition would release more ABA to activate SnRK2 (Fig. 13; Supplementary Table S10). The ABA-responsive element binding factor (ABF) is the main ABA signaling pathway element and positively regulates ABA metabolism. We found that the ABF expression level in NS was higher than in NF after freezing stress, and it was hypomethylated in most regions (Fig. 13; Supplementary Table S10).

The paper showed that brassinolides (BRs) increase plant tolerance to protect plants from damage [63]. BRs are perceived by brassinosteroid insensitive 1 (BRI1), a positive regulator of BR signaling [49]. The cyclin D3 (CYCD3) is an element of the promotive effect of BR on cell division. The result showed that BRI1 and CYCD3 were up-regulated expression and hypermethylated in rapeseed cultivar NS compared to NF after freezing stress (Fig. 12; Supplementary Table S10). Recently, it was shown that the ethylene (ET) signaling pathway transcriptionally inhibits CBF/DREB1 to regulate cold responses of soybean via the action of ethylene insensitive3 (EIN3), contributing to poor cold tolerance [64]. Ethylene receptors (ETRs) as the receptor of ET and negatively regulate the ET signaling pathway [68]. The study showed that the expression levels of EIN3 and ETR2 were lower in NS than in NF, and the methylation level was positively correlated with expression (Fig. 13; Supplementary Table S10). Overall, the phytohormones exert important function in plant freezing tolerance, and DNA methylation is involved to regulates related gene expression.

## Conclusion

Overall, we have provided the first systematic exploration of winter rapeseed's global DNA methylation patterns under freezing stress and investigated several novels and important DMEGs and pathways related to its freezing tolerance mechanism of winter rapeseed. DNA methylation under freezing stress regulated many metabolisms associated with winter rapeseed tolerance.

## Methods

### Plant materials and growth conditions

In this experiment, two winter rapeseed cultivars with different freezing tolerance 17NS57 (NS, freezing-resistant cultivar, with more than 90% overwinter survival rate at − 26 °C) and NQF24 (NF, freezing-sensitive cultivar, with 0% overwinter survival rate below − 10 °C), were provided by the Gansu Agricultural University (Lanzhou, China) [15, 16]. The pot experiment was carried out in a greenhouse. Seeds were germinated in a culture dish on filter papers wetted with deionized water and maintained at 22 ± 1 ℃ until the emergence of the cotyledon. Seedlings of two cultivars were transferred to pots (5L) filled with a 3:1 mixture of nutritional soil and vermiculite and were grown in an illumination incubator under normal conditions (22/20 ℃, day/night temperature, 16 h/8 h light/dark cycle). When the seedlings reached the five leaves stage, they were transferred to the chamber offering freezing treatment at -4 ℃ for 12 h (Treatment 1, t_1_), and 24 h (Treatment 2, t_2_), while the control group (control, t_0_) was maintained in normal condition. After treatment, the third fully expanded leaf from the top of the plant was collected, frozen in liquid nitrogen, and stored at − 80 °C for subsequent analysis.

### DNA and RNA extraction and library construction

DNA from the leaves of six samples was separately extracted using the QIAGEN genomic DNA extraction kit, following the manufacturer’s protocol. Then using Illumina’s standard DNA methylation analysis protocol and a DNA Methylation-Gold Kit constructed libraries. Total RNA was extracted from the leaves of six samples containing three biological replicates using the Spectrum™ Plant Total RNA Kit, mRNA was enriched by Oligo (dT) beads, the enriched mRNA was reverse transcripted into cDNA, and sequencing libraries were generated using QiaQuick PCR extraction kit following the manufacturer’s instructions. For whole-genome bisulfite sequencing (WGBS) and RNA sequencing, the libraries were sequenced on the Illumina Hiseq TM 2500 platform by Gene Denovo BiotechnologyCo (Guangzhou, China).

### Mapping reads to the reference genome

After sequencing, the raw reads were filtered to remove reads containing more than 10% of unknown nucleotides and low-quality reads containing more than 40% of low-quality (Q-value ≤ 20) bases. The obtained clean reads were mapped to the rapeseed reference genome using BSMAP software (version 2.90) by default [69]. Then a custom Perl script was used to call methylated cytosines, and the methylated cytosines were tested with the correction algorithm described in Lister [70]. The methylation level was calculated based on methylated cytosine percentage in the whole genome, in each chromosome, and in different regions of the genome for each sequence context (CG, CHG, and CHH). The methylation profile at flanking 2 kb regions and gene body (or transposable elements) was plotted based on the average methylation levels for each window to assess different methylation patterns in different genomic regions.

### Identification of differentially methylated regions

Differential DNA methylation between the two cultivars at each locus was determined using Pearson's chi-square test (χ^2^) in methyl Kit (version 1.7.10) [71]. To identify DMRs between two cultivars, the minimum read coverage to call a methylation status for a base was set to 4. A sliding-window approach with a 200-bp window sliding at 100-bp intervals was used to identify DMRs. DMRs for each sequence context (CG, CHG, and CHH) according to different criteria: 1) For CG, numbers of GC in each window ≥ 5, the absolute value of the difference in methylation ratio ≥ 0.25, and q ≤ 0.05; 2); For CHG, numbers in a window ≥ 5, the absolute value of the difference in methylation ratio ≥ 0.25, and q ≤ 0.05; 3); For CHH, numbers in a window ≥ 15, the absolute value of the difference in methylation ratio ≥ 0.15, and q ≤ 0.05; 4) For all C, numbers in a window ≥ 20, the absolute value of the difference in methylation ratio ≥ 0.2, and q ≤ 0.05. Genes overlapping with significant DMRs for at least 1 bp in the flanking 2 kb regions and gene body were defined as differentially methylated genes (DMGs), which be used in subsequent analyses.

### RNA sequencing and data analysis

Transcriptome sequencing was performed from the same materials as methylation sequencing containing three biological replicates. The reads containing adapters, reads containing poly-N, and low-quality reads from the raw data were removed using the Trimmomatic software (Bolger et al., 2014). The short reads alignment tool Bowtie2 was used for mapping reads to the ribosome RNA (rRNA) database. The rRNA mapped reads were removed. The remaining reads or unmapped reads were aligned to the rapeseed reference genome (GCA_000686985.2) using TopHat2 (version 2.0.9) [72]. The expression level was normalized by calculating the fragments per kilobase of exon model per million mapped fragments (FPKM) value. To identify differentially expressed genes across samples or groups, the edgeR package (http://www.r-project.org/) was used. All significantly altered genes were identified using the criteria of a *p*-value < 0.001 and a value of |log2foldchange|≥ 2.

### Functional annotation and analysis of DMEGs

To analyze the correlation between altered DNA methylation patterns and gene expression, we examined the overlap between the DMGs and DEGs of two cultivars, named differentially methylated regions related to differentially expressed genes (DMEGs). Then Gene Ontology (GO) enrichment analysis and Kyoto Encyclopedia of Genes and Genomes (KEGG) pathway enrichment analysis were conducted for DMEGs.

### Validation of DMEGs by quantitative qRT-PCR

Total RNA was extracted from the leaves of each sample using the Steadypure Plant RNA Extraction Kit. The first-strand cDNA was synthesized using RNA, according to the manufacturer’s instructions of Evo M-MLV RT Premix for Qrt-PCR, and qRT-PCR amplification reactions were performed using an SYBR® Green Premix Pro Taq HS qPCR Kit. The 2^−ΔΔCT^ method was used to calculate the gene expression, the actin gene (Bra028615) was used as the reference gene for normalization of gene expression, and each sample was replicated three times. The gene names and specific primer sequences were detailed in Supplementary Table S1.

## Supplementary Information

**Additional file 1. Table S1.1** The qPCR rimers used in this study. **Table S1.2** The effective coverage rates of all chromosomes of all samples. **Table S1.3** Number of differentially methylated regions identified in CG, CHG, and CHH contexts between two cultivars of rapeseed. **Table S1.4 **Distribution of methylation levels in different chromosomes.

**Additional file 2. Table S2.1** The DMRs between two cultivars under CG context in control. **Table S2.2** The DMRs between two cultivars under CHG context in control. **Table S2.3** The DMRs between two cultivars under CHH context in control. **Table S2.4** The DMRs between two cultivars under CG context in treatment1. **Table S2.5** The DMRs between two cultivars under CHG context in treatment1. **Table S2.6** The DMRs between two cultivars under CHH context in treatment1. **Table S2.7** The DMRs between two cultivars under CG context in treatment2. **Table S2.8** The DMRs between two cultivars under CHG context in treatment2. **Table S2.9** The DMRs between two cultivars under CHH context in treatment2.

**Additional file 3. Table S3.1** The DMGs between two cultivars under CG context in control. **Table S3.2** The DMGs between two cultivars under CHG context in control. **Table S3.3** The DMGs between two cultivars under CHH context in control. **Table S3.4** The DMGs between two cultivars under CG context in treatment1. **Table S3.5** The DMGs between two cultivars under CHG context in treatment1. **Table S3.6** The DMGs between two cultivars under CHH context in treatment1. **Table S3.7** The DMGs between two cultivars under CG context in treatment2. **Table S3.8** The DMGs between two cultivars under CHG context in treatment2. **Table S3.9** The DMGs between two cultivars under CHH context in treatment2.

**Additional file 4.** **Table S4.1** The data of RNA-seq in two cultivars of rapeseed. **Table S4.2** Number of differentially expression genes between two cultivars of rapeseed. **Table S4.3** Correlation analysis between the three replicates of RNA-seq all libraries.

**Additional file 5. Table S5.1** The DEGs between two cultivars in control. **Table S5.2** The DEGs between two cultivars in treatment1. **Table S5.3** The DEGs between two cultivars in treatment2.

**Additional file 6. Table S6.1** The DMEGs between two cultivars under CG context in control. **Table S6.2** The DMEGs between two cultivars under CHG context in control. **Table S6.3** The DMEGs between two cultivars under CHH context in control. **Table S6.4** The DMEGs between two cultivars under CG context in treatment1. **Table S6.5** The DMEGs between two cultivars under CHG context in treatment1. **Table S6.6** The DMEGs between two cultivars under CHH context in treatment1. **Table S6.7** The DMEGs between two cultivars under CG context in treatment2. **Table S6.8** The DMEGs between two cultivars under CHG context in treatment2. **Table S6.9** The DMEGs between two cultivars under CHH context in treatment2. 

**Additional file 7. Table S7.1** GO classification annotation of 1732DMEGs. **Table S7.2** KEGG pathways analysis of 1732 DMEGs.

**Additional file 8. Table S8.1** Identification of differentially expressed transcription factors for DMEGs. **Table S8.2** Result of TFs between two cultivars in treatment1. **Table S8.3** Result of TFs between two cultivars in treatment2. **Table S8.4** The correlation coefficient between the expression level and the methylation level of the gene encoding the transcription factor. **Table S8.5** Result of kinases between two cultivars in treatment1. **Table S8.6** Result of kinases between two cultivars in treatment2.

**Additional file 9. Table S9.1** Data of RT-qPCR. **Table S9.2** Data of RT-qPCR and RNA-seq.

**Additional file 10. Table S10.1 **Result of DMEGs involved in phosphate metabolism under treatment1. **Table S10.2** Result of DMEGs involved in phosphate metabolism under treatment2. **Table S10.3** Result of DMEGs involved in lipid signal under treatment1. **Table S10.4** Result of DMEGs involved in lipid signal under treatment2. **Table S10.5** Result of DMEGs involved in carbon fixation under treatment1.** Table S10.6** Result of DMEGs involved in carbon fixation under treatment2. **Table S10.7** Result of DMEGs involved in sugar metabolism under treatment1. **Table S10.8** Result of DMEGs involved in sugar metabolism under treatment2. **Table S10.9** Result of DMEGs encoded peroxidase and peroxisome enzymes under treatment1. **Table S10.10** Result of DMEGs encoded peroxidase and peroxisome enzymes under treatment2. **Table S10.11** Result of DMEGs involved in flavonoid biosynthesis under treatment1. **Table S10.12** Result of DMEGs involved in flavonoid biosynthesis under treatment2. **Table S10.13** Result of DMEGs involved in plant hormone signal transduction under treatment1. **Table S10.14** Result of DMEGs involved in plant hormone signal transduction under treatment2.

**Additional file 11. Figure S1. **Distribution of methylation levels in different genomic functional regions of two rapeseed cultivars. A, CG context; B, CHG context; C, CHH context.

**Additional file 12. Figure S2. **Distribution of methylation levels in different transposable elements of two rapeseed cultivars. A, CG context; B, CHG context; C, CHH context.

## Data Availability

The sequenced transcriptome raw data and Whole-genome bisulfite sequencing raw data have been deposited to the SRA at NCBI with the accession number PRJNA685002.

## Abbreviations

DMRs: Differentially methylated regions
DMGs: Differentially methylated genes
DEGs: Differentially expressed genes
DMEGs: DEGs associated with DMGs
RNA-Seq: Ribonucleic acid sequencing
qRT-PCR: Quantitative Real-Time PCR
WGBS: Whole-genome bisulfite sequencing
MSAP: Methylation sensitive amplification polymorphism
MSP: Methylation specific PCR
TE: Transposable elements
rRNA: Ribosome RNA
GO: Gene Ontology
KEGG: Kyoto Encyclopedia of Genes and Genomes
