# Supplementary figures and images for: Integrated methylome and transcriptome analysis unravel the cold tolerance mechanism in winter rapeseed(Brassica napus L.)
Source: BMC Plant Biol. 2022 Aug 26;22:414. doi: 10.1186/s12870-022-03797-1 (PMC9414130; doi:10.1186/s12870-022-03797-1)

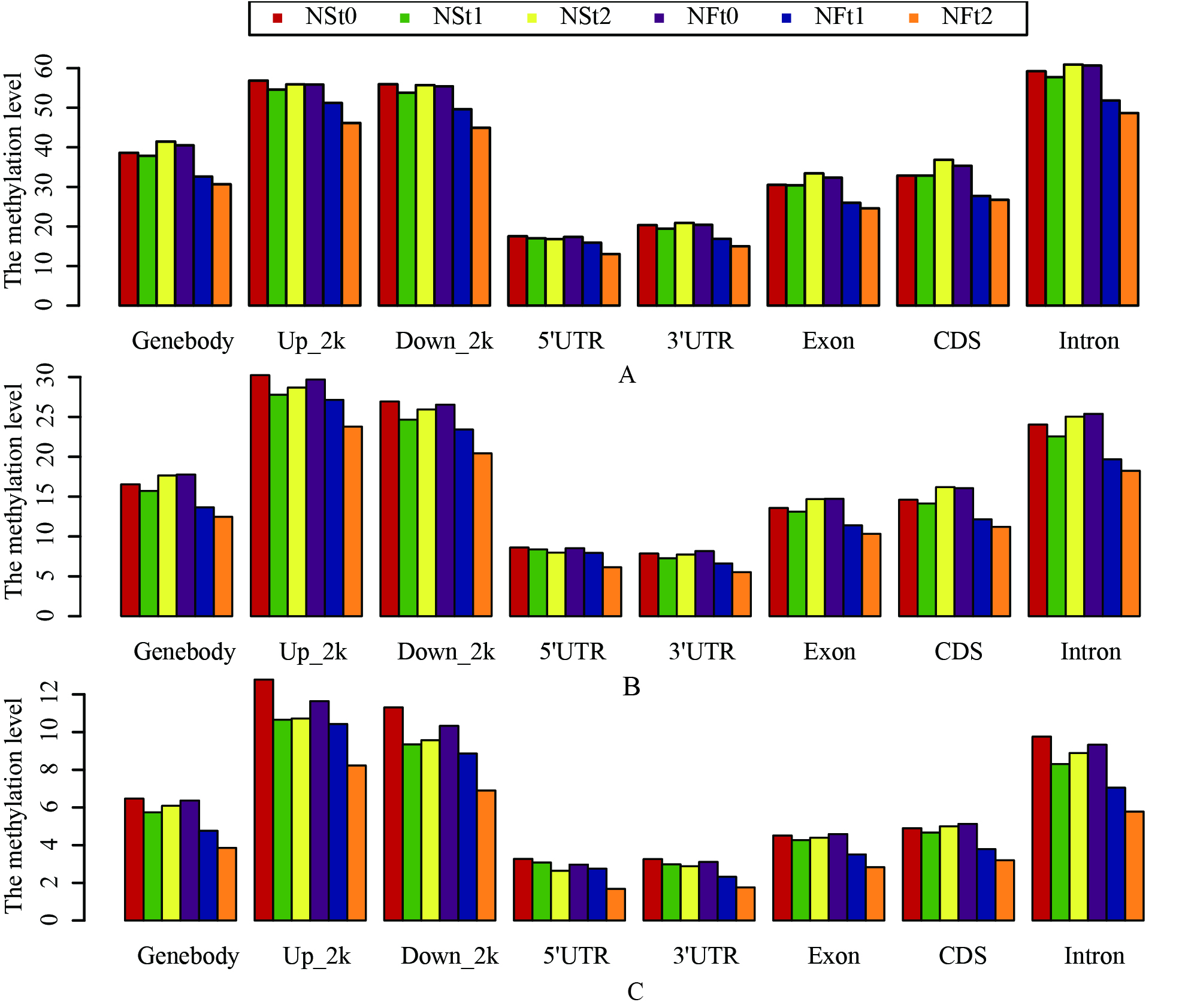

Supplement: Supplementary file 11 — Additional file 11. Figure S1. Distribution of methylation levels in different genomic functional regions of two rapeseed cultivars. A, CG context; B, CHG context; C, CHH context. [file 12870_2022_3797_MOESM11_ESM.jpg]

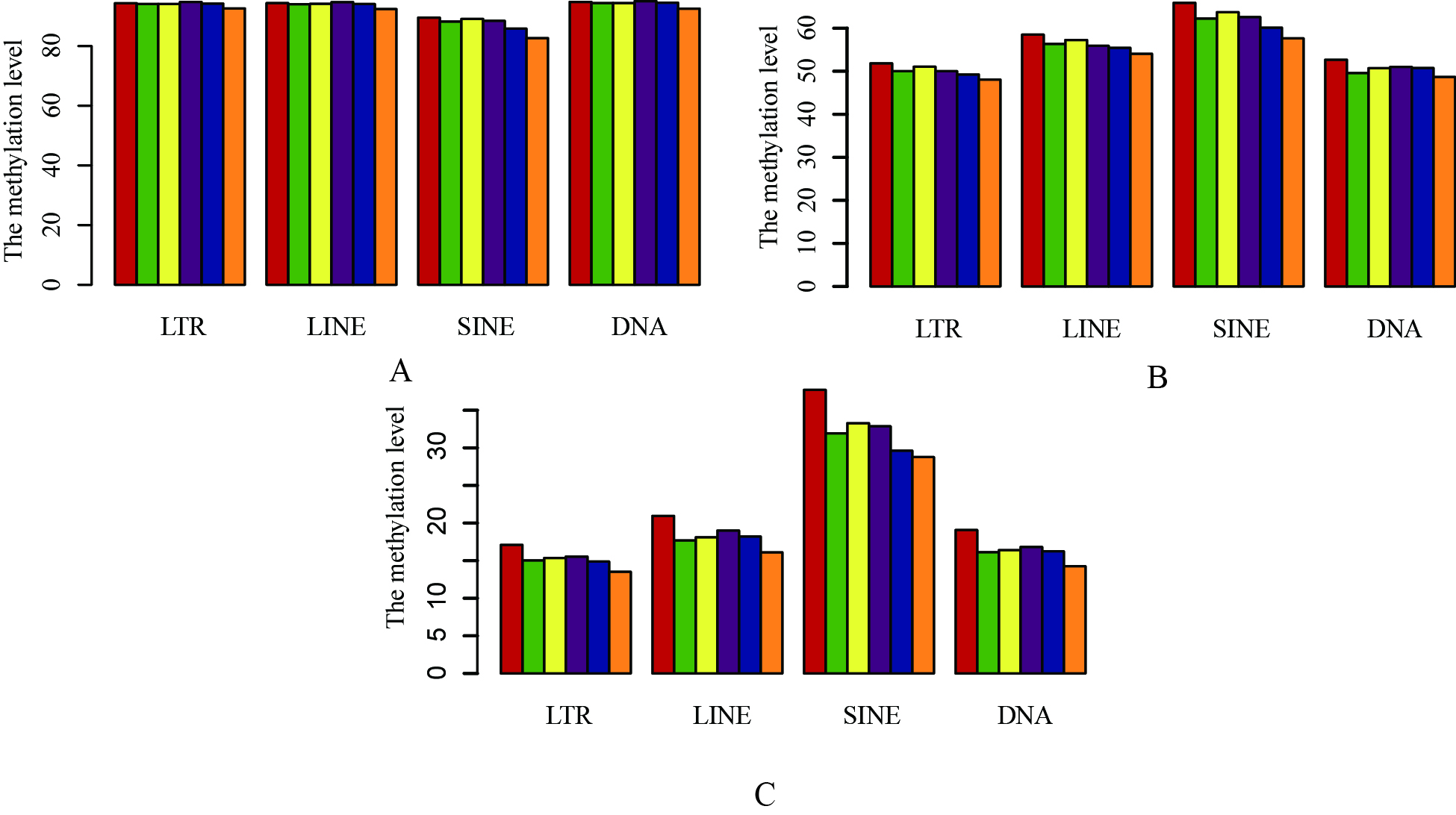

Supplement: Supplementary file 12 — Additional file 12. Figure S2. Distribution of methylation levels in different transposable elements of two rapeseed cultivars. A, CG context; B, CHG context; C, CHH context. [file 12870_2022_3797_MOESM12_ESM.jpg]
